# Reunion of Australasian Possums by Shared SINE Insertions

**DOI:** 10.1093/sysbio/syac025

**Published:** 2022-03-15

**Authors:** Liliya Doronina, Charles Y Feigin, Jürgen Schmitz

**Affiliations:** Institute of Experimental Pathology (ZMBE), University of Münster, Von-Esmarch-Str. 56, D-48149 Münster, Germany; Department of Molecular Biology, Princeton University, 119 Lewis Thomas Laboratory, Washington Road, Princeton, NJ 08544-1014, USA; School of BioSciences, The University of Melbourne, BioSciences 4, Royal Pde, Parkville, VIC 3010, Australia; Institute of Experimental Pathology (ZMBE), University of Münster, Von-Esmarch-Str. 56, D-48149 Münster, Germany

## Abstract

Although first posited to be of a single origin, the two superfamilies of phalangeriform marsupial possums (Phalangeroidea: brushtail possums and cuscuses and Petauroidea: possums and gliders) have long been considered, based on multiple sequencing studies, to have evolved from two separate origins. However, previous data from these sequence analyses suggested a variety of conflicting trees. Therefore, we reinvestigated these relationships by screening $\sim$200,000 orthologous short interspersed element (SINE) loci across the newly available whole-genome sequences of phalangeriform species and their relatives. Compared to sequence data, SINE presence/absence patterns are evolutionarily almost neutral molecular markers of the phylogenetic history of species. Their random and highly complex genomic insertion ensures their virtually homoplasy-free nature and enables one to compare hundreds of shared unique orthologous events to determine the true species tree. Here, we identify 106 highly reliable phylogenetic SINE markers whose presence/absence patterns within multiple Australasian possum genomes unexpectedly provide the first significant evidence for the reunification of Australasian possums into one monophyletic group. Together, our findings indicate that nucleotide homoplasy and ancestral incomplete lineage sorting have most likely driven the conflicting signal distributions seen in previous sequence-based studies. [Ancestral incomplete lineage sorting; possum genomes; possum monophyly; retrophylogenomics; SINE presence/absence.]

The proposed marsupial suborder Phalangeriformes is comprised of two superfamilies: Phalangeroidea (brushtail possums, cuscuses, and pygmy possums) and Petauroidea (Leadbeater’s, ringtail, honey, and feathertail possums and petaurid gliders). More than three decades have passed since morphological investigations of the marsupial auditory region supported the monophyly of Phalangeriformes (e.g., by a fusion of the ectotympanic bone with other bones of the skull [Flannery 1987; Springer and Woodburne 1989]). Subsequent single-copy DNA hybridization studies further supported the morphology-based phylogeny (Springer and Kirsch 1991; Kirsch et al. 1997).

However, molecular data collected since then did not significantly support possum monophyly. Rather, the relationship was consistently challenged by evidence for a close affinity between Macropodiformes (kangaroos, wallabies, bettongs, potoroos, and rat kangaroos) and one or the other of its constituent possum superfamilies (Phalangeroidea$+$Macropodiformes [Beck 2008; Phillips and Pratt 2008; Meredith et al. 2008, 2009], Petauroidea$+$Macropodiformes [Meredith et al. 2011; Mitchell et al. 2014; May-Collado et al. 2015]). The latter grouping was even supported by a recent large-scale analysis of 1550 exonic loci (Duchêne et al. 2018). Notably, these data also included some evidence for ancestral incomplete lineage sorting (ILS). ILS is due to polymorphic signals passing successive speciation events, followed by subsequent random fixation. ILS within Phalangeroidea, Petauroidea, and Macropodiformes (together with koalas and wombats, forming the order Diprotodontia) is not unexpected, considering their short ancestral speciation times (a period of $<$3 million years [Mitchell et al. 2014]). Together with nucleotide homoplasy, ILS might have driven the conflicting signal distributions seen in previous molecular sequence studies and is termed hemiplasy (Avise and Robinson 2008).

Retrotransposed elements (REs) are increasingly used as alternative cladistic presence/absence markers to resolve challenging phylogenies. A transcribed retrotransposon is copied via reverse transcription and simultaneously inserted into a random genomic location. Its large number of possible insertion sites across the genome (character states) are flanked by unique target site duplications that can be used to precisely detect orthologous insertion positions in related species. The presence of an inserted RE in the exact same genomic location in two species indicates their phylogenetic closeness compared to the absence in distant species (Fig. 1, left). Several important phylogenetic relationships have been resolved based on RE presence/absence patterns: Salem et al. (2003) and Roos et al. (2004) in primates; Nishihara et al. (2005) in afrotherians, Kriegs et al. (2006) in early placental lineages; Churakov, Sadasivuni et al. (2010) in rodents; Suh et al. (2011) in birds; Doronina et al. (2015) in carnivorans; and Doronina et al. (2017a) in laurasiatherians.

**Figure 1.**
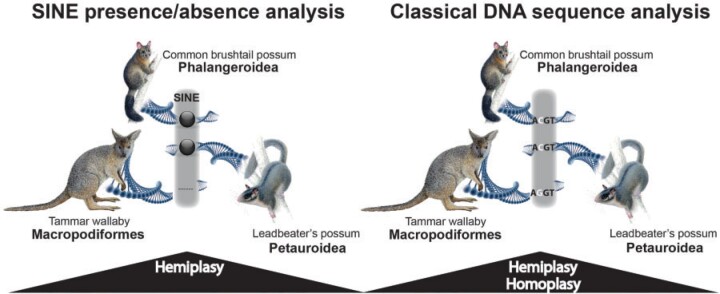
Comparison of SINE presence/absence and classical DNA sequence analyses. Left: The common brushtail possum and the Leadbeater’s possum share a diagnostic SINE (black ball) at an orthologous genomic position (pictured as gray area). The SINE was inherited from a shared common ancestor of Phalangeroidea plus Petauroidea and is absent (dashes) in Macropodiformes and all outgroup representatives. Right: The classical DNA sequence analysis compares orthologous conserved sequences that can be variably exposed to homoplasy depending on selection and drift. Both marker systems are equally exposed to hemiplasy as a result of ILS.

A number of high-throughput genome screening tools to extract and analyze diagnostic RE markers are now publicly available: (i) “TinT” is used to inspect the timeframe of RE insertion waves (Churakov, Grundmann et al. 2010), (ii) “GPAC” (genome presence/absence compiler) serves to extract genome-wide diagnostic REs from multiway genome alignments (alignments of multiple genomes; Noll et al. 2015) and (iii) “2-n-way” enables the genome-wide extraction of diagnostic REs from combinations of pairs of assembled genomes (Churakov et al. 2020a). The automated, computational extraction of orthologous RE presence/absence regions from multiple species with alternating reference genomes (e.g., via the multicomparative 2-n-way suite; Churakov et al. 2020a) provides an extensive and reliable genome data source for phylogenomic reconstructions in cases where other marker systems often fail. Evaluating the statistical significance of RE presence/absence data is also crucial. It can be accomplished with two specially designed statistical tools: KKSC for three-species/lineage comparisons (Kuritzin et al. 2016) and 4-LIN for four-species/lineage comparisons (Churakov et al. 2020b), both of which can identify cases of ILS.

Short interspersed elements (SINEs) are nonautonomous REs derived from cellular RNAs but do not encode a functional reverse transcriptase protein. Instead, they rely on autonomous mobile transposons, especially long interspersed elements (LINEs) to repetitively retrotranspose and integrate more or less randomly back into the genome. Uniquely in mammals, their efficient LINE1-mediated, reverse transcription, and random genomic insertion provide hundreds of diagnostic signals that, once irreversibly fixed in the ancestral genome, unmistakably mark all descendent lineages as monophyletic (Hillis 1999). As indicated for bird phylogeny (Matzke et al. 2012), REs might increasingly accumulate and fix during episodes of reduced population sizes (bottlenecks) followed by rapid radiation. Combined with their nearly homoplasy-free nature (Doronina et al. 2019), SINEs are reliable extraordinal markers, especially in problematic phylogenetic anomaly zones (Fig. 1).

The first large-scale phylogenetic SINE analyses in marsupials were conducted by Nilsson et al. (2010) and Zemann et al. (2013). These studies revealed an extensive distribution of SINEs, especially WSINEs and WALLSIs, throughout major marsupial diversifications and, for example, supported a single shared origin of all Australasian marsupials. In Feigin et al. (2018), we used SINE presence/absence patterns to examine the phylogenetic position of the thylacine, the extinct marsupial Tasmanian tiger. Although we did detect a small number of conflicting markers that could be explained by ILS, there were a significant number of phylogenetic diagnostic markers that placed the thylacine at the first diversification point of Dasyuromorphia.

With Australasian possums, we move to another exciting group of marsupials with a suggested paraphyletic affiliation and relatively short internodes (Duchêne et al. 2018). In so doing, we present the first genome-wide analysis of SINE presence/absence markers that significantly supports the long refuted monophyly of possums. We hold that these new analyses justify a phylogenetic reclassification of the Australasian possums within Diprotodontia, which will undoubtedly revive discussions and investigations into the evolution, biogeography, and biology of this largest group of marsupials.

## Materials and Methods

### Genomes

We generated pairwise alignments of multiple marsupial genomes and extracted potential phylogenetically informative orthologous SINE loci to analyze their exact presence/absence states, as well as the sequence phylogeny of their 1 kb flanking regions. To investigate the possible relationships between Phalangeriformes–Macropodiformes, we performed multidirectional screens of the genomes of representative species in the Phalangeroidea (common brushtail possum; *Trichosurus vulpecula*; unpublished sequencing project; mTriVul1.pri, NCBI, https://www.ncbi.nlm.nih.gov/), Petauroidea (Leadbeater’s possum; *Gymnobelideus leadbeateri*; LBP_v1, NCBI), and Macropodiformes (tammar wallaby; *Notamacropus eugenii*; me-1k.fasta, DNA Zoo) as well as the outgroup koala (*Phascolarctos cinereus*; phaCin_unsw_v4.1, NCBI) for SINE elements.

### RepeatMasking

SINE masking was performed for all target and query species genomes with RepeatMasker (https://www.repeatmasker.org/RepeatMasker/) and the integrated standard “Metatheria” library (Supplementary Data S2 available on Dryad at https://doi.org/10.5061/dryad.xpnvx0kgk). Using fastCOEX (Doronina et al. 2017b), we extracted the RepeatMasker coordinates of nearly complete ($<$10 nt truncations on 5$^\prime$- and 3$^\prime$-ends) WSINE1, WSINE1a, WALLSI1, and WALLSI1a elements along with their transposed element (TE)-sparse flanks ($<$50$\%$ TEs in 500 nt flanks).

### Whole-Genome Alignments

2-n-way is a software suite that generates 2-way LAST or LASTZ genome alignments. It combines them in a multicomparative framework to screen for presence/absence patterns of REs (based on their genomic coordinates) at orthologous loci (e.g., see Fig. 1 left; Churakov et al. 2020a). We generated nine pairwise whole-genome alignments in the 2-n-way suite from the genomic sequences of the above representatives of Phalangeroidea, Petauroidea, and Macropodiformes, as well as the outgroup, koala, as follows: (i) common brushtail possum/Leadbeater’s possum, (ii) common brushtail possum/tammar wallaby, (iii) common brushtail possum/koala, (iv) Leadbeater’s possum/common brushtail possum, (v) Leadbeater’s possum/tammar wallaby, (vi) Leadbeater’s possum/koala, (vii) tammar wallaby/common brushtail possum, (viii) tammar wallaby/Leadbeater’s possum, and (ix) tammar wallaby/koala. For multidirectional screens, we first transferred these two-ways to the n-way module of 2-n-way. Then, using the extracted SINE coordinates, we performed three screenings with standard settings and MUSCLE-based optimization to extract aligned sequences of orthologous loci for the following perfect presence/absence patterns: (i) Phalangeroidea ($+$), Petauroidea ($+$), Macropodiformes ($-$) (Fig. 1 left), (ii) Phalangeroidea ($+$), Macropodiformes ($+$), Petauroidea ($-$), and (iii) Petauroidea ($+$), Macropodiformes ($+$), Phalangeroidea ($-$).

### Diagnostic Orthologous SINEs

To verify the presence/absence patterns within each of the three lineages mentioned above, we supplemented these sequences with those from additional representatives of Phalangeroidea, Petauroidea, and Macropodiformes. The following add- itional diprotodontian genome sequences were retrieved from DNA Zoo (https://www.dnazoo.org/): Phalangeroidea: *Phalanger gymnotis* (pg-2k.fasta); Pet- auroidea: *Pseudochirops corinnae* (Pseudochirops_ corinnae_HiC.fasta), *Pseudochirops cupreus* (Pseudo- chirops_cupreus_HiC.fasta), *Pseudocheirus occidentalis* (Pseudocheirus_occidentalis_HiC.fasta); Macropodi- formes: *Macropus rufus* (mr-2k.fasta), *Macropus fuliginosus* (mf-2k.fasta), *Macropus giganteus* (mg-2k. fasta), *Setonix brachyurus* (Setonix_brachyurus_ HiC.fasta) (Dudchenko et al. 2017, 2018). The common wombat (*Vombatus ursinus*; bare-nosed wombat genome assembly, NCBI) sequence was taken as an alternative outgroup (Supplementary Table S1 available on Zenodo).

LINE1-derived 8-30 nt long target site duplications are an important indicator of element boundaries and were identified and indicated in the marker alignments (Kuritzin et al. 2016; Supplementary Data S1 available on Dryad). Summarizing the selection criteria, we selected WSINEs, and WALLSI1 elements that were: (i) active during the diversification of Australasian marsupials (Nilsson et al. 2010; Zemann et al. 2013), (ii) were nearly full-length SINEs ($<$10 nt truncations), (iii) were flanked by 500 nt sequences that were depleted of other TEs (specifically, $<$50$\%$ TEs) so as to omit nested elements which might complicate clear orthology assignments. Additionally, for loci passing the above criteria, we filtered out any which showed evidence of duplication and applied strict orthology criteria for species comparisons, allowing only identical SINE types inserted in the same orientation, shifted in position by less than 3 nt, whose presence or absence was consistent in two or more representatives per lineage and showed a clear absent state in the outgroup species. The SINE markers that satisfied all these criteria were considered to be phylogenetically informative.

### Genomic Marker Distribution, Statistical Analysis, and Tree Reconstruction

To verify the random genomic locations of markers and thus make sure there was no marker concentration bias, we derived their chromosomal distributions based on the genome assembly of the Tasmanian devil (*Sarcophilus harrisii*, mSarHar1.11, NCBI) via the BLAST genome tool. We evaluated the statistical significance of our presence/absence data using the KKSC insertion significance test (Kuritzin et al. 2016), which assesses presence/absence patterns of 3-lineages for their probability of being different from polytomy. KKSC can distinguish between conflicting signals derived from ILS (random accumulation) or ancestral hybridization (biased accumulation) based on the symmetry or asymmetry of marker numbers supporting alternative tree topologies. The basics of the applied probabilistic model are described by Waddell et al. (1999). The KKSC test is principally similar to the ABBA-BABA test (D statistics) that analyses the presence of introgression in SNP data (single-nucleotide polymorphism; for a review, see Springer et al. 2020).

We built a presence/absence (1/0) SINE matrix (Supplementary Table S2 available on Zenodo at https://doi.org/10.5281/zenodo.5584509) to visualize conflicting signals in a neighbor-net analysis in SplitsTree, version 4.13.1, standard settings (Huson and Bryant 2006) and a Bayesian tree reconstruction (MrBayes 3.2; ctype irreversible, mcmc ngen$=$20000 samplefreq$=$100 printfreq$=$100 diagnfreq$=$1000; Ronquist et al. 2012).

### Flanking Sequence-Based Analyses

To determine the cause of conflicting patterns, we also performed sequence-based phylogenetic analyses of the flanking regions of phylogenetically informative RE insertions by constructing three concatenated alignments from the three different groups of RE markers: (i) Phalangeroidea$+$Petauroidea, (ii) Phalangeroidea$+$Macropodiformes, and (iii) Petauroidea$+$Macropodiformes. We extracted $\sim$1 kb per locus of the RE presence or absence flanking regions and concatenated three data sets (catfasta2phyml.pl; https://github.com/nylander/catfasta2phyml). We then performed maximum phylogenetic parsimony and maximum phylogenetic likelihood analyses (PAUP 4.0; standard settings; Swofford 2002), neighbor-net analysis in SplitsTree (version 4.13.1, standard settings; Huson and Bryant 2006), and Bayesian inference of phylogenetic trees (MrBayes 3.2; lset nst$=$6 rates$=$invgamma, GTR (generalised time reversible) substitution model, mcmc ngen$=$400.000 samplefreq$=$100 printfreq$=$100 diagnfreg$=$1000; Ronquist et al. 2012). Furthermore, we used IQ-TREE2 (Minh et al. 2020a) to calculate gene and site concordance values (Minh et al. 2020b) for the flanking DNA sequences after inferring IQ-TREE2 species trees and gene/locus trees under default settings.

### 11,532 Random Genomic Loci Sequence Analysis

Additionally, data comprised of random orthologous, nonoverlapping, nonexonic genomic regions across a set of 13 marsupial species (for species see Supplementary Table S1 available on Zenodo) were collected for further phylogenetic maximum parsimony (MP) and maximum likelihood (ML) analyses. Bedtools (bedtools v2.27.1; Quinlan and Hall 2010) was used to initially extract 150,000 random 2.5 kb genomic loci from the Hi-C version of the koala genome (phaCin_unsw_v4.1_HiC.fasta; DNA Zoo). These regions were filtered such that no two regions were located closer than 10 kb together along a scaffold or overlapped any exon (based on the RefSeq koala gene annotation, which was lifted over to the Hi-C koala genome using *liftoff* v1.6.1 with distance-scaling parameter -d set to 4). To save on computational time, a randomized selection of these loci ($\sim$28,000) were extracted from the genomes of the 12 remaining species using *liftoff* (parameters described above) and then aligned using *mafft* (–localpair –maxiterate 1000 –adjustdirectionaccurately). These alignments were then trimmed to remove alignment columns dominated by gaps and low-quality sequences with trimAl (v1.4.rev22; Capella-Gutiérrez et al. 2009) using the *-strict* setting, which eliminates columns that are outliers in the per-alignment gap and divergence distributions, to exclude regions of missing data or nonorthologous segments (such as those arising from small sequence rearrangements). Any orthologous alignment found to still contain gaps $>$25$\%$ of the total alignment length or composed of 50$\%$ gaps in total were excluded. Finally, the remaining 11,532 loci that passed filtering were concatenated and used as input for subsequent analyses. A phylogenetic MP analysis was performed on the concatenated alignment using PAUP* 4 (4a168; Swofford 2002). Vombatiformes was set as an outgroup, and a search (bandb) was performed with 1000 bootstrap replicates to find the best phylogenetic MP tree. ML phylogenetic analysis was performed using RAxML (version 8.2.12; Stamatakis 2014). We selected the most straightforward default settings and models for sequence-based analyses. For analysis of concatenated alignments, the following parameters were used (-f a -x 58744 -p 58744 -m GTRGAMMA; -f a$=$ rapid bootstrap analysis and search for best-scoring ML tree in one program run -x$=$rapidBootstrapRandomNumberSeed, 58744 -p$=$parsimonyRandomSeed 58744 -m GTRGAMMA$=$GTR $+$ Optimization of substitution rates $+$ GAMMA model of rate heterogeneity) with 1000 bootstrap replicates and the Vombatiformes (koala and wombat) set as the outgroup. We also analyzed the 11,532 alignments in IQ-TREE 2 to calculate gene and site concordance values for all three possible speciation scenarios.

## Results and Discussion

As whole-genome sequences are now available for many marsupial species, we revisited the pre-established possum paraphyletic relationship, testing currently discussed hypotheses for the phylogenetic affiliations within Diprotodontia. To accomplish this, we exploited the highly reliable presence/absence patterns of SINEs (Shedlock et al. 2004; Doronina et al. 2019), analyzing them via strategies to visualize the evolutionary effects of genome-wide ILS or ancestral hybridization (Kuritzin et al. 2016).

LINE1-mediated SINE RNA reverse transcription and endonucleolytic genomic insertion are exceptionally efficient in therian lineages such as marsupials. In the therian ancestor, LINE1 lost specificity and is now capable of coretrotransposing any polyA-tailed sequences such as SINEs. Most therian orders host specific SINE elements that primarily originated from polyadenylated tRNAs or 7SL RNAs. They may propagate in up to more than a million individual copies, making them potentially useful as phylogenetic clade markers. Other taxa contain similarly suitable RE types, for example, LINE-derived elements in birds like the chicken repeat CR1.

However, as an upper limit, because of the accumulation of mutations, for clades that evolved more than 100 Ma, it is challenging to determine exact RE insertion orthology (Kriegs et al. 2006). At the lower limit, clades separated during the last $\sim$2 million years carry unfixed polymorphic insertions that may misdirect phylogenetic reconstruction (Kuritzin et al. 2016).

The investigated possums separated $\sim$50 Ma (Duchêne et al. 2018) in conjunction with the high activity of marsupial SINEs at that time (Nilsson et al. 2010; Zemann et al. 2013), making them a particularly suitable group for retrophylogenomic reconstructions (genome-wide analysis of retrotransposon presence/absence patterns for phylogenetic inference).

**Figure 2.**
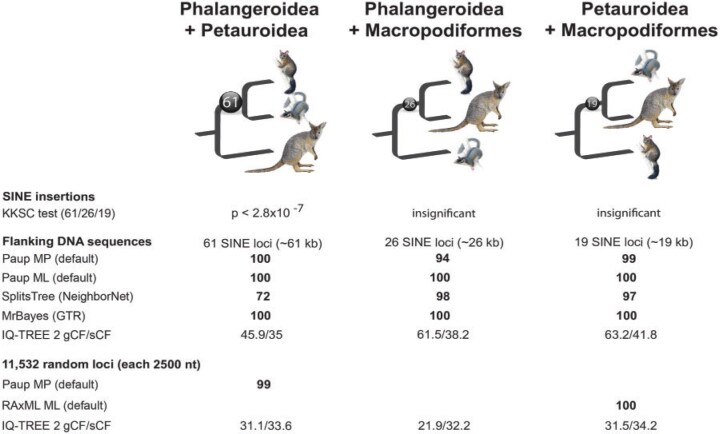
Compilation of phylogenetic signals for presence/absence SINE markers, their flanking sequence regions, and a random genome sequence data set from 11,532 loci. Sixty-one SINE insertions (black ball) provide significant evidence to merge Phalangeroidea and Petauroidea (KKSC test, $P< 2.8 \times 10^{-7}$). Sequence flanks of the three sets of conflicting SINE insertions (61/26/19) imply considerable support for the respective topologies of the SINEs. The 11,532 randomly selected sequence loci (each 2500 nt) revealed two conflicting tree topologies depending on the method applied (MP vs. ML). The IQ-TREE 2 gene concordance factor (gCF) and site concordance factor (sCF) for flanking and the 11,532 random sequences are shown below the bootstrap results. Bootstrap values are represented in bold.

**Figure 3.**
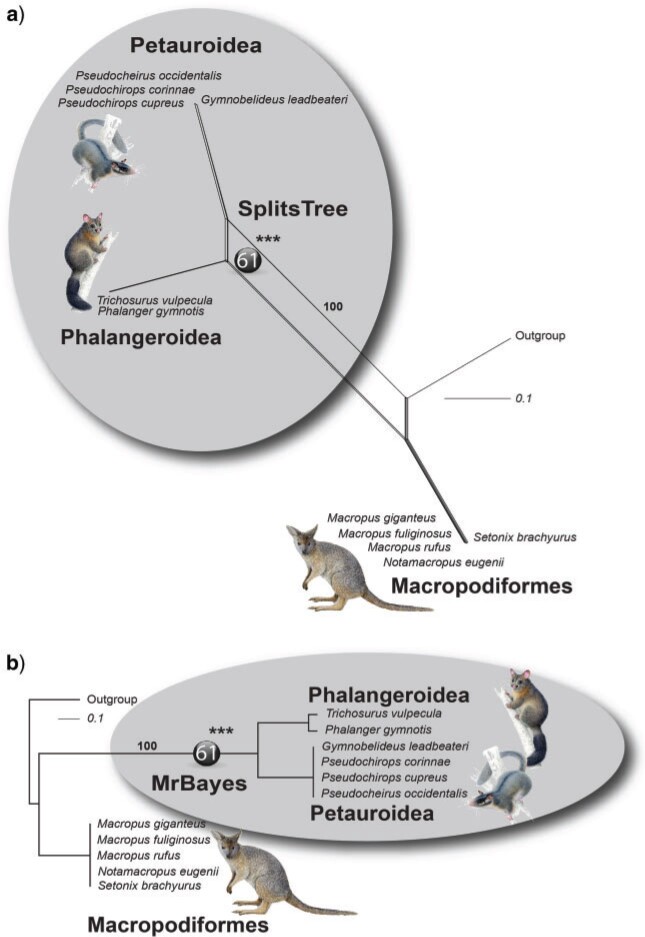
Phylogenetic reconstructions of informative SINE presence/absence loci. a) SplitsTree analysis. Sixty-one SINE loci (black ball) are mapped on the common branch of Phalangeroidea and Petauroidea, a branch substantiated by 100$\%$ bootstrap support. b) Bayesian inference of the presence/absence loci revealed a common origin for Phalangeroidea and Petauroidea by 100$\%$ bootstrap resampling.

We used the 2-n-way method (Churakov et al. 2020a) to align and screen representative genomes of these groups for relevant, phylogenetically informative SINEs. From respectively 167,441, 996,890, and 126,275 SINEs identified by the RepeatMasker, we extracted and analyzed 29,165 loci with nearly full-length elements and TE-deficient flanks ($<$50$\%$ TEs) from the common brushtail possum (representing Phalangeroidea), 154,300 from the genome of the Leadbeater’s possum (Petauroidea), and 10,576 from the tammar wallaby (Macropodiformes). We then analyzed these patterns to test the following possible hypotheses: (i) Phalangeriformes monophyly, (ii) Phalangeroidea–Macropodiformes sister-group relationships, and (iii) the Petauroidea–Macropodiformes affiliation. The 2-n-way screening revealed 506 potential informative markers regarding the diprotodontian relationships. After manual analysis for orthology (see essential criteria in Materials and Methods section), we compiled 106 phylogenetically informative markers whose loci were randomly distributed over the reference genome of the Tasmanian devil (a well-annotated outgroup to the diprotodont marsupials), in intergenic regions (65 markers) and introns (41 markers) (Supplementary Table S1 available on Zenodo). From the 106 diagnostic genomic loci, 61 SINE markers were present in Phalangeroidea and Petauroidea genomes and absent in Macropodiformes, 26 were present in Phalangeroidea and Macropodiformes but not in Petauroidea, and 19 were present in Petauroidea and Macropodiformes but absent in Phalangeroidea genomes (Fig. 2, Supplementary Table S1 and Supplementary Data S1 available on Zenodo and Dryad). The statistical KKSC test (Kuritzin et al. 2016) revealed highly significant support for the Phalangeriformes monophyly (p $<$ 2.8$\,\times\,$10$^{-7}$) and no evidence for ancestral hybridization (Supplementary Fig. S1 available on Zenodo). Neighbor-net and Bayesian inference analyses of the presence/absence patterns showed the same tree topology as that determined by KKSC (Fig. 3).

To recognize a potential mosaic structure of genomic recombination units comprising the diagnostic SINE markers supporting each of the three opposing hypotheses (supported by 61, 26, and 19 markers), we performed sequence-based phylogenetic analyses of the regions flanking these loci (concatenated sequences for each of the three topologies, Supplementary Data S3–S5 available on Dryad). We used phylogenetic MP, ML, neighbor-net, and Bayesian inference analyses of these flanking regions. We found close to a 100$\%$ fit between our SINE markers and flanking sequence phylogeny (Fig. 2). These results provide the first evidence for a mosaic structure of the genome in marsupials, with recombination units inherited from different ancestral lineages, a pattern previously shown in primates (Pääbo 2003) and laurasiatherians (Doronina et al. 2017a). Different genomic regions exhibit conflicting phylogenetic signals. Therefore, investigating a small number of loci or those from only individual recombination regions may fail to reveal the true diprotodontian phylogeny. Moreover, we confirmed that the loci we analyzed (SINE loci and random genomic sequences) were randomly distributed throughout the entire genomes. It should be noted that our diagnostic SINEs did not cotransfer any flank regions during their original retrotransposition, as evidenced by their consistent presence/absence patterns (Supplementary Data S1 available on Dryad).

We further explored phylogenetic sequence signals across the genome by performing sequence-based analyses of the 11,532 concatenated, random noncoding loci, each 2500 nt long (Supplementary Data S6 available on Dryad). Interestingly, we observed a conflicting pattern between two analysis approaches, with MP strongly supporting phalangeriform monophyly (consistent with SINE presence/absence patterns; Supplementary Fig. S2 available on Zenodo) and ML analyses strongly supporting a Petauroidea–Macropodiformes sister-group relationship. This indicates sequence-inherited inconsistencies that are irrelevant in SINE presence/absence analyses. The IQ-TREE 2 derived gene and site concordance factors (gCF and sCF) (Fig. 2) diverge from the strong but opposite bootstrap support (MP supporting Phalangeriformes monophyly, ML supporting Petauroidea$+$Macropodiformes). That indicates, at least for the sCF of the 11,532 concatenated random noncoding sequences, that the sequence signals may be at an area of parameter space where ILS might mislead the ML analyses (Kubatko and Degnan 2007) compared with the MP analyses (Mendes and Hahn 2018).

In contrast to previous sequence-based studies, the presence/absence patterns of SINEs identified in our genome-wide searches strongly supported the monophyly of possums to exclude Macropodiformes, revisiting the early studies (Springer and Woodburne 1989; Springer and Kirsch 1991; Kirsch et al. 1997). That our SINE presence/absence findings contradict those of most previous sequence analyses (e.g., Phillips and Pratt 2008; Meredith et al. 2008; Mitchell et al. 2014) may possibly be explained by the small number of nuclear loci that were previously examined, which made many prior analyses particularly vulnerable to the confounding effects of ILS in such rapidly radiating groups, as well as to homoplasy present in sequence-based analyses. RE presence/absence patterns, by contrast, are for all practical purposes almost free of parallel insertions, precise deletions, and nonallelic gene conversion-caused homoplasy (Doronina et al. 2019, 2021). If ILS does not completely overlay the phylogenetic signal, whole-genome RE data can find the correct species tree, while sequence-based analyses may remain inconclusive. In the case when ILS does completely overlay the phylogenetic signal, as in many neoavian bird lineages (Jarvis et al. 2014; Suh et al. 2015), or there was insufficient RE activity during critical periods, REs will also fail to resolve such trees and may lead to hard polytomy.

Interestingly, the phylogenetic relationships significantly supported by our data contradict the only available large-scale genomic analysis of diprotodontian genomes (Duchêne et al. 2018). This study predominantly supported two alternative tree reconstructions: Petauroidea$+$Macropodiformes (1046 loci) and Phalangeroidea$+$Macropodiformes (504 loci). Although we also found RE markers supporting both hypotheses, they were both in lower, nonsignificant numbers. Our KKSC test (Kuritzin et al. 2016) revealed that genome-wide, randomly distributed conflicting markers occurred most probably due to ILS rather than ancestral hybridization. We should note that Duchêne et al. (2018) analyzed only exonic sequence regions, whose evolution is strongly affected by natural selection. Notably, they revealed a highly asymmetrical distribution of trees, assigning them to ILS. The pressure of selection on coding regions may indeed lead to an asymmetric tree distribution in the presence of ILS (He et al. 2019), and together with homoplastic phylogenetic signals, may confound phylogenetic reconstructions using sequence-based approaches. In contrast, our SINE presence/absence markers from both introns and intergenic regions integrated most likely in the absence of any directional or purifying selection. Even the occasional functionally adopted REs represent neutral markers because their original insertion place was chosen without selective forces (for functional REs, see Schrader and Schmitz 2019). Thus, our whole-genome, multidirectional RE screening provides the first highly significant evidence for the common origin of all Australasian possums.

With the accumulation of new genome sequences, phylogenetic reconstructions based on RE presence/absence patterns are receiving increasingly more attention. One should keep in mind, however, that multidirectional screening (equally testing all possible tree topologies) does require comparable, high-quality genome assemblies and marker-by-marker manual verification of true orthology. For Mammalia and Sauropsida (reptiles and birds), a high and continuous RE activity facilitates their versatility as clade markers. Nevertheless, presence/absence RE data is as equally challenged by ILS as are all other marker systems and cannot always resolve particular phylogenetic problems, especially in groups with rapidly radiating species such as neoavian birds, which are notorious for exhibiting conflicting markers (Jarvis et al. 2014; Suh et al. 2015). Whereas sequence-based analyses can be troubled by homoplasy, the virtually homoplasy-free RE presence/absence patterns provide superb phylogenetic signals and can directly visualize the fallout from ILS. The simple character states (0 for absence and 1 for presence) enable a transparent interpretation without complex variation models but cannot reveal more than shared ancestral insertion information.

On the other hand, the distributions of REs in populations and species follow the same rules as those for other marker systems. They are equally sensitive to population bottlenecks and rapid radiation. Their wave-like temporal activity renders them unsuitable for dating splits and restricts their main power to reconstruct complex speciation scenarios in combination with sequence data. We and others have applied screenings for diagnostic presence/absence REs within and among nearly all orders of mammals and some other vertebrates. The scarcity of studies outside of vertebrates may signal (i) a lack of good-quality genome assemblies, (ii) that elements were just not active at the critical time of shared ancestry, or (iii) that divergence times were too deep to recognize clear orthology. However, their present use, enabled by just recently completed genome information and sufficient numbers of embedded SINE elements active 50 Ma, did allow us to finally lay to rest the previous erroneously supposed paraphyly of these possum groups and, instead, to demonstrate the common ancestry of all living possums.

## Supplementary Material

Data available from the Dryad Digital Repository: https://doi.org/10.5061/dryad.xpnvx0kgk. Supporting information is available from Zenodo: https://doi.org/10.5281/zenodo.5584509.
